# An assessment of primary health care costs and resource requirements in Kaduna and Kano, Nigeria

**DOI:** 10.3389/fpubh.2023.1226145

**Published:** 2023-12-19

**Authors:** Yewande Ogundeji, Hamza Abubakar, Uche Ezeh, Tijjani Hussaini, Nelson Kamau, Eliza Love, Rodrigo Muñoz, Paul Ongboche, Marjorie Opuni, Damian G. Walker, Colin Gilmartin

**Affiliations:** ^1^Health Strategy and Delivery Foundation, Abuja, Nigeria; ^2^Kaduna State Primary Health Care Board, Kaduna, Nigeria; ^3^Kano State Primary Health Care Management Board, Kano, Nigeria; ^4^Management Sciences for Health, Arlington, VA, United States; ^5^Sistemas Integrales, Santiago, Chile; ^6^Independent Consultant, Lausanne, Switzerland

**Keywords:** cost, Kaduna, Kano, minimum service package, Nigeria, primary health care, strategic planning, universal health coverage

## Abstract

**Introduction:**

The availability of quality primary health care (PHC) services in Nigeria is limited. The PHC system faces significant challenges and the improvement and expansion of PHC services is constrained by low government spending on health, especially on PHC. Out-of-pocket (OOP) expenditures dominate health spending in Nigeria and the reliance on OOP payments leads to financial burdens on the poorest and most vulnerable populations. To address these challenges, the Nigerian government has implemented several legislative and policy reforms, including the National Health Insurance Authority (NHIA) Act enacted in 2022 to make health insurance mandatory for all Nigerian citizens and residents. Our study aimed to determine the costs of providing PHC services at public health facilities in Kaduna and Kano, Nigeria. We compared the actual PHC service delivery costs to the normative costs of delivering the Minimum Service Package (MSP) in the two states.

**Methods:**

We collected primary data from 50 health facilities (25 per state), including PHC facilities—health posts, health clinics, health centers—and general hospitals. Data on facility-level recurrent costs were collected retrospectively for 2019 to estimate economic costs from the provider’s perspective. Statewide actual costs were estimated by extrapolating the PHC cost estimates at sampled health facilities, while normative costs were derived using standard treatment protocols (STPs) and the populations requiring PHC services in each state.

**Results:**

We found that average actual PHC costs per capita at PHC facilities—where most PHC services should be provided according to government guidelines—ranged from US$ 18.9 to US$ 28 in Kaduna and US$ 15.9 to US$ 20.4 in Kano, depending on the estimation methods used. When also considering the costs of PHC services provided at general hospitals—where approximately a third of PHC services are delivered in both states—the actual per capita costs of PHC services ranged from US$ 20 to US$ 30.6 in Kaduna and US$ 17.8 to US$ 22 in Kano. All estimates of actual PHC costs per capita were markedly lower than the normative per capita costs of delivering quality PHC services to all those who need them, projected at US$ 44.9 in Kaduna and US$ 49.5 in Kano.

**Discussion:**

Bridging this resource gap would require significant increases in expenditures on PHC in both states. These results can provide useful information for ongoing discussions on the implementation of the NHIA Act including the refinement of provider payment strategies to ensure that PHC providers are remunerated fairly and that they are incentivized to provide quality PHC services.

## 1 Introduction

Nigeria’s government aims to strengthen its health system, especially its primary health care (PHC) system, ensuring all Nigerians have access to “quality, effective, efficient, equitable, accessible, affordable, and comprehensive health care services” (1). In 2011, the National Council on Health launched the Primary Health Care Under One Roof (PHCUOR) policy to address the fragmented nature of PHC (2). This policy consolidated PHC under the authority of the State Primary Health Care Development Agency/Board (SPHCDA/B) with the Local Government Health Authority (LGHA) overseeing PHC delivery at health facilities and in communities, and reporting to the SPHCDA/B (3). The PHCUOR policy also introduced the Minimum Service Package (MSP) (Supplementary Figure S1). This essential service package, adaptable by the states, aims to ensure that residents receive a basic level of health services at PHC facilities, which include health posts, primary health clinics, and primary health care centers (4).

Access to quality PHC services remains an important challenge in Nigeria, with only 44% of the population using essential health services (5). The PHC system grapples with multiple issues: inefficient supply chains, inadequate drugs, medical supplies, and equipment at health facilities, and poor health worker performance manifested by high levels of absenteeism and low levels of competence and productivity (6). Fewer than half of Nigeria’s public PHC facilities maintain a consistent supply of essential drugs, and many lack basic utilities like electricity (7). As a result, the private sector plays a major role in health service provision, delivering more than 50% of the country’s health services (2). Many residents’ first contact with the health system is via Patent and Proprietary Medicine Vendors (PPMVs) (8).

Efforts to expand and enhance PHC services in Nigeria are also constrained by the low level of government expenditure on health (2, 9, 10). In 2020, just 4% of government expenditure went to health, far short of the 15% Abuja Declaration target (11). When considering the proportion of government expenditure on PHC, it constitutes only 14% of overall expenditure on PHC (11). Most government spending on PHC comes from local government areas (LGAs), funded primarily through federal government revenues and value-added taxes (7). Out-of-pocket (OOP) expenses dominate health financing, accounting for 75% of health spending (11), with 16% of Nigerians incurring catastrophic health expenses, consuming over 10% of their household income (5). Established by the National Health Act of 2014, the Basic Health Care Provision Fund (BHCPF) is a critical initiative by Nigeria that aims to address the country’s health financing gaps (12, 13). This fund aims to improve PHC delivery by channeling investments directly into PHC infrastructure, human resources, medicines, and commodities, and ensuring free PHC services for the poor.

A variety of health insurance schemes exist in Nigeria (2). The legislation of the National Health Insurance Scheme (NHIS), adopted in 1999, led to the establishment of the Formal Sector Social Health Insurance Programme in 2005. With the decentralization of the NHIS in 2016 and the guidelines for the operationalization of the BHCPF (13), most states introduced their own insurance schemes. In addition, community-based and private health insurance options are also available. Yet, by 2018, only 3% of Nigerians aged 15–49 were insured (14). A notable limitation of the NHIS legislation was that participation in health insurance schemes was voluntary (2). The National Health Insurance Authority (NHIA) Act was passed in May 2022 to “promote, regulate, and integrate health insurance schemes in Nigeria” (15). The NHIA Act mandates health insurance for all citizens and legal residents and prescribes a basic health service package that all health insurance schemes must cover across states (15, 16). The law also stipulates the establishment of a Vulnerable Group Fund—to be funded in part by the BHCPF—designed to cover premiums for vulnerable populations, including children under five years, pregnant women, the older adult, individuals with disabilities, and those living in poverty (15, 16).

Channeling a greater share of resources and services towards PHC facilities and ensuring that these facilities provide quality services as efficiently as possible is critical to improving access to essential health services in Nigeria and advancing towards universal health coverage (UHC). The ongoing development of the NHIA operational guidelines (17) presents a unique opportunity in this regard and a comprehensive understanding of the costs associated with primary health service delivery is paramount. Understanding the cost of PHC services can assist in formulating provider payment strategies that ensure that providers are remunerated fairly and that they are incentivized to provide quality services (18–22). Under the NHIS, primary health services were procured from accredited public and private healthcare providers through capitation payments of N750/US$ 2.4 per member per quarter for a designated benefit package which largely overlaps with the MSP (Supplementary Figure S2) (18). A criticism of the NHIS was that capitation payments for primary health services—determined using actuarial methods (18)—were too low (18, 23, 24).

This study sought to determine the costs of providing PHC services at public facilities in Kaduna and Kano, Nigeria. We compared these actual or real-world costs with the normative or theoretical costs associated with implementing established MSPs. This comparison shed light on the financial gap that must be addressed to ensure comprehensive, universal PHC coverage in the two states. Specifically, the study: (1) assessed actual costs of services at PHC facilities—health posts, primary health clinics, and primary health care centers—in Kaduna and Kano; (2) calculated the normative costs of delivering the services included in the MSPs in both states; (3) identified the financial gap between the actual and normative costs considering only PHC services provided at PHC facilities; and (4) estimated the financial gap between the actual and normative costs considering PHC services delivered at both PHC facilities and general hospitals.

## 2 Materials and methods

### 2.1 Study setting

Kaduna and Kano, located in northeastern Nigeria, were purposively selected for this study. These states are densely populated, with populations of 8.5 million in Kaduna and 13 million in Kano as of 2016 (25). Both states have high poverty levels; 50–60% of their populations live in monetary poverty, surpassing the national average of 40% (26). When considering multidimensional poverty, which takes into account access to basic infrastructure and services, 70–80% of residents in these states are affected, compared to almost 50% nationally (26). In terms of health indicators, Kaduna and Kano habitually fall below national averages. Neonatal and under-five mortality rates are 63 and 187 per 1,000 live births in Kaduna and 37 and 164 in Kano, compared to national rates of 39 and 132 (14). The percentage of births attended by skilled health workers is also notably lower in these states—26.5% in Kaduna and 21.5% in Kano—compared to the national average of 43.3% (14). Regarding health financing, while state-specific data for Kano are unavailable, Kaduna predominantly relies on private health care financing—primarily OOP expenditures—which constitutes 80% of the state’s health expenditure (27). State-level data on health insurance coverage are not available (14).

### 2.2 Costing tool

We used the open-access Primary Health Care Costing, Analysis, and Planning (PHC-CAP) Tool (28) for our cost estimates of PHC services in Kaduna and Kano. The PHC-CAP Tool is an activity-based costing tool in Microsoft Excel, which enables users to calculate recurrent actual and normative costs of PHC services provided in a geographic area. Actual costs are calculated using data collected from a sample of health facilities which are expanded to the universe of facilities in the geographic area of interest. Normative costs represent the resources essential for efficient, quality service delivery (29). Normative costs are calculated using standard treatment protocols (STPs) developed for all services in a PHC service package, with the estimated populations in need of each service based on population size and expected service provision given estimated disease incidence and prevalence rates as well as utilization rates for promotional and preventive services. The difference between the two costs represents the financial resource gap. In accordance with the World Health Organization’s PHC Measurement Framework and Indicators (30), key metrics that can be analyzed in the PHC-CAP Tool include inpatient and outpatient services per clinical staff, daily service output per clinical staff, cost per input, cost per service, total cost, and cost per capita—contingent on data availability. Aside from Nigeria, the PHC-CAP Tool has also been used in five additional countries, including Ethiopia (31) and Kenya (32).

### 2.3 Data collection

For the actual PHC costing, we used multi-stage sampling to select the sample of health facilities. Initially, local government areas (LGAs) were selected from Kaduna (12 of 23) and Kano (20 of 43), based on 2019 PHC service provision, 2019 health data reporting, geographical distribution of facilities, facility accessibility, and perceived security risk (Supplementary Figure S3). Subsequently, 25 public health facilities in each state were selected, stratified by LGA and type of facility, using the Master Health Facility Lists last updated in 2018 (Supplementary Table S1). In Kaduna, this included 1 health post, 9 health clinics, 10 health centers, and 5 general hospitals. Kano’s sample consisted of 7 health posts, 8 health clinics, 6 health centers, and 4 general hospitals. The categorization of facility types in the Master Facility List sometimes differed from what was observed during data collection, with some health posts, health clinics, and health centers upgraded or downgraded. We therefore grouped health posts, health clinics, and health centers into one “PHC facilities” category in accordance with government guidelines (4). General hospitals did not change classifications.

We collected 2019 data on both the outputs of PHC services as delivered at health facilities and the recurrent inputs employed to generate these services, and their respective prices. The data collected spanned four input categories: clinical and non-clinical labor, drugs, medical supplies, and operational inputs like electricity and water. We considered inputs irrespective of funding source, be it local, state, or federal government, donors, and OOP for drugs and medical supplies at facilities to estimate costs. The focus was on recurrent costs, excluding capital costs due to time and data limitations. Data limitations also led us to exclude above-facility costs, such as training and supervision by state and federal administrations.

Researchers from the Health Strategy and Delivery Foundation (HSDF) trained data collectors on the data collection tools and methods. Following the training, the data collection teams participated in a pilot at two health facilities in Nasarawa, Kaduna and Wudil, Kano. HSDF researchers set up control rooms in each state to supervise data collection in real-time, providing oversight and support to data collectors as needed. Data collection at health facilities took place from May to June 2021 in Kano and from October to November 2021 in Kaduna. Following data collection, the collected data were compiled into separate datasets for each state. Once compiled, these datasets underwent thorough data cleaning and the data were analyzed for irregularities, including incomplete entries and outliers. The HSDF team further investigated all detected anomalies.

Data availability varied by facility and state. In Kaduna, facility registers yielded partial data on quantities and prices of drugs and medical supplies. These data were then refined using data from the state drug funds and insights from state officials. Kano, however, lacked facility and state records of quantities of drugs and medical supplies for 2019 and beyond. Drug and medical supply expenditures for health facilities in Kano were derived by estimating the quantities of drugs dispensed and medical supplies used in our sample of facilities and estimating their unit costs, in consultation with facility heads and state officials. Drug costs represent market prices (33) and a suggested 16% increase to cover distribution, logistics, and supply chain expenses, as recommended by state officials. In both states, staff composition and salaries were collected from the human resource records at the health facilities, supplemented by interviews with facility heads and state budget data. Operational cost estimates drew from financial records at the health facilities, interviews, and state budget data. 2019 inpatient and outpatient service data for government-run health posts, health clinics, health centers, and general hospitals in Kaduna and Kano states were sourced from the Nigeria District Health Information System 2 (DHIS2), covering both our sample and the universe of health facilities in the states.

The study considered all recurrent inputs and service outputs at PHC facilities—health posts, primary health clinics, and primary health care centers—as these are designated primary health facilities (4). In the case of general hospitals, only the costs of PHC units were considered. Cost data for general hospitals in Kaduna were excluded from this analysis because of challenges encountered in clearly isolating PHC-specific costs. To approximate the actual PHC costs within general hospitals for Kaduna, we used the cost data from PHC units in Kano’s general hospitals as a proxy. We recognize that limiting to PHC units contributes to an underestimate of the true costs of delivering PHC in these settings. Using Kano data as a proxy for Kaduna is also a limitation of this analysis, given the discernible differences in the cost structures of PHC facilities between the two states.

### 2.4 Costing approach

As per the Lancet Global Health Commission on financing PHC, for actual costing, PHC services are defined as the services delivered at PHC facilities (34). To calculate the annual actual costs of PHC services as delivered at each facility surveyed, we aggregated the 2019 labor, drug, medical supply, and operational costs. Because of difficulties in apportioning the expenditure data collected on outpatient visits and inpatient days, these were apportioned assuming one inpatient day equated 4.4 outpatient visits at PHC facilities and 3.9 outpatient visits at general hospitals (35)^1^—assumptions which are consistent with previous costing studies (36). To calculate the cost per patient, total costs at health facilities were divided by weighted service outputs, where weighted service outputs were equal to the sum of outpatient visits (OP) and 4.4 times inpatient (IP) days at PHC facilities and the sum of OP visits and 3.9 times IP days at general hospitals.

To determine the overall PHC cost for Kaduna and Kano, we used two different approaches to first expand the total annual actual costs from sampled PHC facilities to all public PHC facilities providing outpatient services as recorded in the DHIS2 for 2019 and then to expand from sampled PHC facilities and general hospitals to all public PHC facilities and general hospitals. In the first estimation method (estimate 1), expansion factors were derived based on service utilization. In the second estimation method (estimate 2), expansion factors were calculated based on numbers of health facilities. Both methods presumed that costs at the health facilities surveyed reflected statewide averages. Per capita actual costs for Kaduna and Kano were determined by dividing the total cost of each estimate for each state by the respective populations. This approach of approximating total actual PHC costs in a geographic area by extrapolating from a sample of health facilities has been employed in several previous studies (31, 32, 37, 38).

For normative costs, we costed the national MSP which the states have adopted (39, 40). While the MSPs for both states shared most services, they differed in target coverage levels for some services. A team of clinicians developed STPs for all 103 services included in the MSPs. These protocols specified average facility visits per service per year, staff time per service, required drugs and diagnostics per services, all priced accordingly. The population needing each service was determined using state demographic data and data on incidence and prevalence rates as well as utilization rates for promotional and preventive services. Incidence and prevalence rates were obtained from national sources or the 2019 Global Burden of Disease (GBD) dataset for Nigeria (41). To factor in indirect costs including the costs of non-clinical labor and operational inputs in the normative costs, we applied an overhead derived from our facility survey data. We calculated indirect cost rates for each facility type by dividing indirect costs by the total cost. We did not consider potential efficiency gains through economies of scale and scope in the normative cost estimates.

The financial resource gaps for PHC services in Kaduna and Kano were calculated as the difference between actual and normative costs (31, 32, 37, 38). We calculated resource gaps using actual estimates 1 and 2 described above. Our baseline financial resource gaps assumed that the entirety of the population in Kaduna and Kano would exclusively use public sector primary health facilities. However, recognizing that over half of health services in Nigeria are provided in the private sector (2), we also estimated resource gaps assuming 50 and 75% of the population used public sector services.

All costs are expressed in United States Dollars (US$). The exchange rate applied was 316 Nigerian Naira (NGN) per US$ (42).

## 3 Results

### 3.1 Characteristics of sampled PHC facilities

There were significant differences in staffing levels at the sampled PHC facilities in Kaduna and Kano (Table 1), pointing to possible deviations from staffing guidelines. Notably, the PHC facilities in Kaduna employed three times as many clinical staff as those in Kano. The volume of services delivered also varied between states (Table 1). While the quantity of outpatient services was comparable in Kaduna and Kano, PHC facilities in Kaduna reported inpatient days but no inpatient days were reported in Kano. The clinical staff caseload also differed by state (Table 1), but the average number of daily services handled was notably low in both states, suggesting high levels of inefficiency. Staff at Kaduna’s PHC facilities managed an average of two services per day while their counterparts in Kano handled four services.

**Table 1:** Characteristics of sampled PHC facilities, Kaduna and Kano, 2019.

	Kaduna	Kano
Number of sampled PHC facilities	21	21
Number of staff		
Clinical staff		
Mean	15	5
Median	13	4
Range	1–47	1–23
Non-clinical staff		
Mean	4	5
Median	3	5
Range	0–17	0–15
Total staff		
Mean	19	10
Median	14	8
Range	1–56	2–30
Number of services		
Outpatient visits per year		
Mean	2,483	3,006
Median	2,086	1,924
Range	608–7,553	425–13,034
Inpatient days per year		
Mean	114	0
Median	5	0
Range	0–576	0
Number of services per clinical staff per day		
Mean	2	4
Median	1	3
Range	0–4	1–23

The number of services per clinical staff per day was determined by dividing weighted service outputs for 2019 in a facility by the clinical staff count times working days [178 days according to Okoroafor et al., (58)]. Weighted service outputs is the sum of outpatient visits (OP) and 4.4 times inpatient (IP) days, where 4.4 is the ratio of the cost per IP day in primary hospitals (PPP I$ 21.69) over the cost per OP visit in bed-equipped health centers (PPP I$ 4.93), based on unit cost data from WHO-CHOICE (35).

### 3.2 Actual costs at sampled PHC facilities

The average annual service delivery costs at the PHC facilities sampled in Kaduna were somewhat lower than those in Kano, although PHC facilities in Kaduna exhibited a wider range of costs (Table 2). Whereas clinical labor costs and medical supply costs were higher at PHC facilities in Kaduna, drug costs and indirect costs were higher at PHC facilities in Kano.

**Table 2:** Total costs in sampled PHC facilities, Kaduna and Kano, US$, 2019.

	Kaduna	Kano
Number of sampled PHC facilities	21	21
Total costs		
Mean	240,332	277,219
Median	182,427	270,155
Range	8,777–1,544,734	86,375–565,142
Total clinical labor costs		
Mean	65,313	24,167
Median	41,531	20,794
Range	2,141–354,314	6,185–103,075
Total drug costs		
Mean	110,028	140,859
Median	77,536	152,064
Range	5,445–779,267	51,335–268,942
Total supply costs		
Mean	48,888	27,412
Median	24,794	23,500
Range	1,091–395,855	3,189–57,869
Total indirect costs		
Mean	13,967	49,360
Median	10,064	44,867
Range	51–50,966	5,979–126,667

Clinical labor includes labor of doctor, nurse/midwife, community health officer, CHEW, JCHEW, CHIPS, health attendant, lab technician, pharmacy technician, environmental officer with clinical duties, pharmacist, lab scientist, nutritionist, health educator, CHIPS, other labor. Indirect costs include operational expenses and labor of medical record officer, environmental officer, and support volunteers.

**Figure 1:**
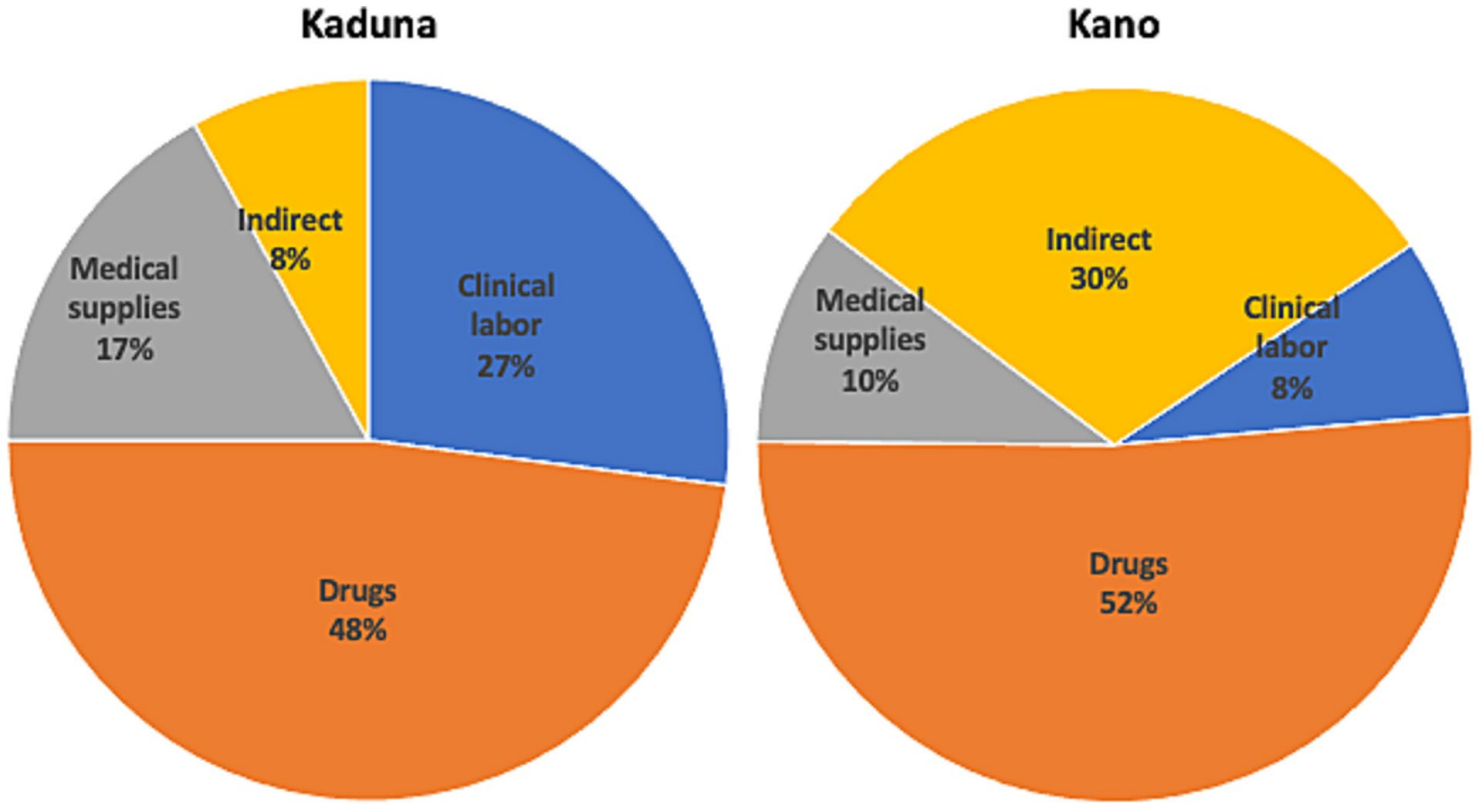
Distribution of actual costs at sampled PHC facilities, Kaduna and Kano, 2019. *Clinical labor* includes labor of doctor, nurse/midwife, community health officer, CHEW, JCHEW, CHIPS, health attendant, lab technician, pharmacy technician, environmental officer with clinical duties, pharmacist, lab scientist, nutritionist, health educator, CHIPS, other labor. *Indirect costs* include operational expenses and labor of medical record officer, environmental officer, and support volunteers.

Clinical labor costs constituted 27% of average annual costs in Kaduna and 8% in Kano (Figure 1). In Kaduna, drug and medical supply costs accounted for 48% and 17% of costs respectively, while in Kano, these categories represented 52% and 10% of costs. Indirect costs accounted for 8% of costs in Kaduna and 30% of costs in Kano.

**Figure 2:**
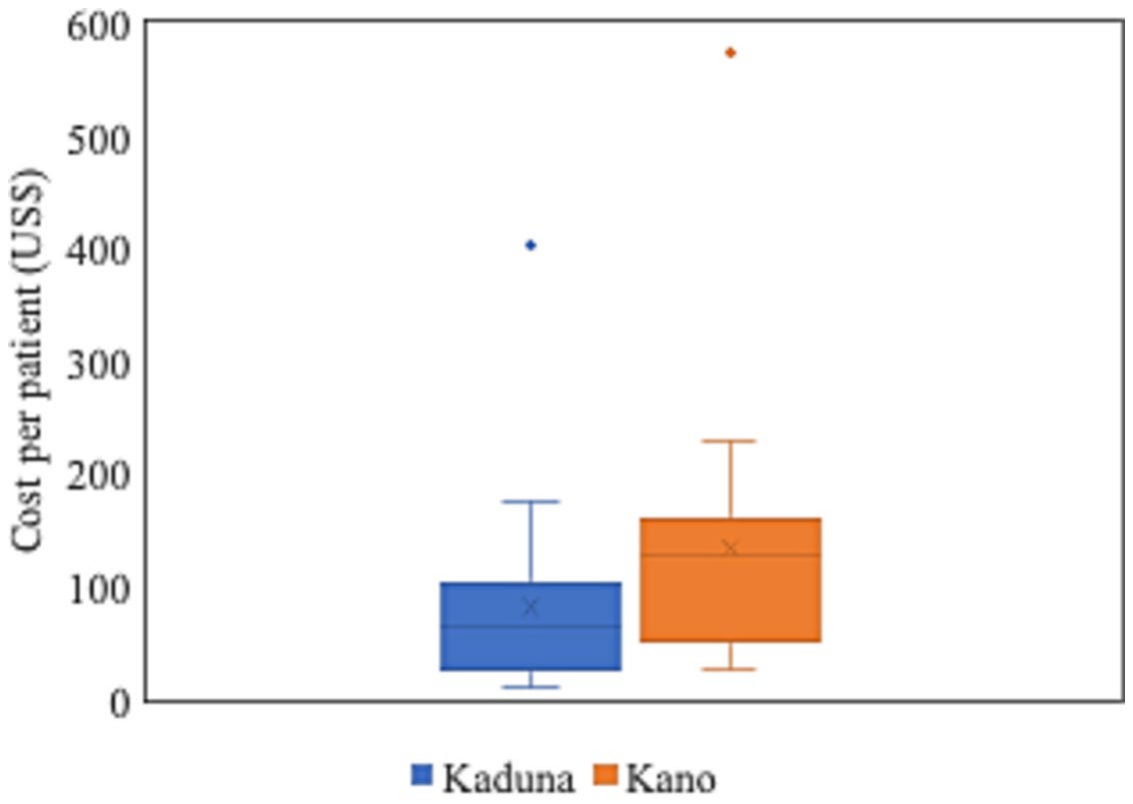
Cost per patient at sampled PHC facilities, Kaduna and Kano, 2019. Cost per patient at each sampled PHC facility calculated by dividing total costs by weighted service outputs. Weighted service output is the sum of outpatient visits (OP) and 4.4 times inpatient (IP) days, where 4.4 is the ratio of the cost per IP day in primary hospitals (PPP I$ 21.69) over the cost per OP visit in bed-equipped health centers (PPP I$ 4.93), based on unit cost data from WHO-CHOICE (35).

Assessing the average costs per patient at the sampled PHC facilities revealed very high costs per patient in both states, suggesting significant inefficiencies (Figure 2). Average costs per patient were US$ 82.2 at PHC facilities in Kaduna and US$ 133.6 at Kano’s PHC facilities with costs per patient ranging from US$ 9.4 to US$ 403.9 at PHC facilities in Kaduna and US$ 28.2 to US$ 574.5 at PHC facilities in Kano.

### 3.3 Actual and normative costs at statewide PHC facilities

Table 3 shows the total annual actual costs of PHC services as delivered at PHC facilities, extrapolated from the sampled PHC facilities to all state PHC facilities. In Kaduna, these costs ranged between US$ 173.7 million to US$ 257.3 million, depending on the estimation method employed. In Kano, the costs ranged from US$ 227.5 million to US$ 291.6 million. Notably, all actual cost estimates were substantially lower than the projected normative costs—US$ 412.9 million in Kaduna and US$ 707.8 million in Kano—which accounted for the full package of services as per state MSPs and expanded service utilization based on population need (Supplementary Table S2).

**Table 3:** Statewide actual and normative PHC costs by input at PHC facilities only, Kaduna and Kano, 2019.

	Kaduna	Kano
Actual costs (US$ million)		
Estimate 1		
Total	173.7	227.5
Clinical labor	47.6	22.7
Drugs	80.2	132.5
Medical supplies	35.6	25.8
Indirect	10.2	46.4
Estimate 2		
Total	257.3	291.6
Clinical labor	70.5	29.1
Drugs	118.8	169.9
Medical supplies	52.8	33.1
Indirect	15.1	59.5
Normative costs (US$ million)		
Total	412.9	707.8
Clinical labor	100.9	118.3
Drugs	178.4	290.4
Medical supplies	109.4	154.6
Indirect	24.2	144.5
Number of PHC facilities in network	1,080	1,207
Population (million)	9.2	14.3

Actual cost estimates calculated as follows: estimate 1, total actual costs in sample expanded to entire state based on service utilization; estimate 2, total actual costs in sample expanded to entire state based on numbers of facilities. Clinical labor in actual costs includes labor of doctor, nurse/midwife, community health officer, CHEW, JCHEW, CHIPS, health attendant, lab technician, pharmacy technician, environmental officer with clinical duties, pharmacist, lab scientist, nutritionist, health educator, CHIPS, other labor. Clinical labor in normative costs includes labor of doctor nurse/midwife, community health officer, CHEW, JCHEW, and CHIPS. Indirect costs in actual costs include operational expenses and labor of medical record officer, environmental officer and support volunteers. Indirect costs in normative costs are imputed based on the proportion observed in actual costs.

With a population of 9.2 million, the actual per capita costs in Kaduna ranged from US$ 18.9 to US$ 28 compared to the normative per capita cost of US$ 44.9 (Table 4). Similarly, in Kano, with a population of 14.3 million, the actual per capita costs ranged from US$ 15.9 to US$ 20.4 compared to the normative per capita cost of US$ 49.5. These gaps suggest considerable underfunding of PHC services at PHC facilities in both states.

**Table 4:** Statewide actual and normative PHC costs per capita and corresponding gaps by input, at PHC facilities only, Kaduna and Kano US$, 2019.

	Kaduna				Kano			
	Actual cost per capita		Normative cost per capita	Gap	Actual cost per capita		Normative cost per capita	Gap
	Estimate 1	Estimate 2			Estimate 1	Estimate 2		
Clinical labor	5.2	7.7	11.0	3.3–5.8	1.6	2.0	8.3	6.2–6.7
Drugs	8.7	12.9	19.4	6.5–10.7	9.3	11.9	20.3	8.4–11.0
Medical supplies	3.9	5.7	11.9	6.2–8.0	1.8	2.3	10.8	8.5–9.0
Indirect	1.1	1.6	2.6	1.0–1.5	3.2	4.2	10.1	5.9–6.9
Total	18.9	28.0	44.9	16.9–26.0	15.9	20.4	49.5	29.1–33.6
Number of PHC facilities in network				1,080				1,207
Population (million)				9.2				14.3

Actual cost per capita estimates calculated as follows: estimate 1, total actual cost in sample expanded to entire state based on service utilization, then divided by total state population; estimate 2, total actual costs in sample expanded to entire state based on numbers of facilities, then divided by total state population. Clinical labor in actual costs includes labor of doctor, nurse/midwife, community health officer, CHEW, JCHEW, CHIPS, health attendant, lab technician, pharmacy technician, environmental officer with clinical duties, pharmacist, lab scientist, nutritionist, health educator, CHIPS, other labor. Clinical labor in normative costs includes labor of doctor nurse/midwife, community health officer, CHEW, JCHEW, and CHIPS. Indirect costs in actual costs include operational expenses and labor of medical record officer, environmental officer and support volunteers. Indirect costs in normative costs are imputed based on the proportion observed in actual costs.

### 3.4 Actual and normative costs at statewide PHC facilities and general hospitals

**Figure 3:**
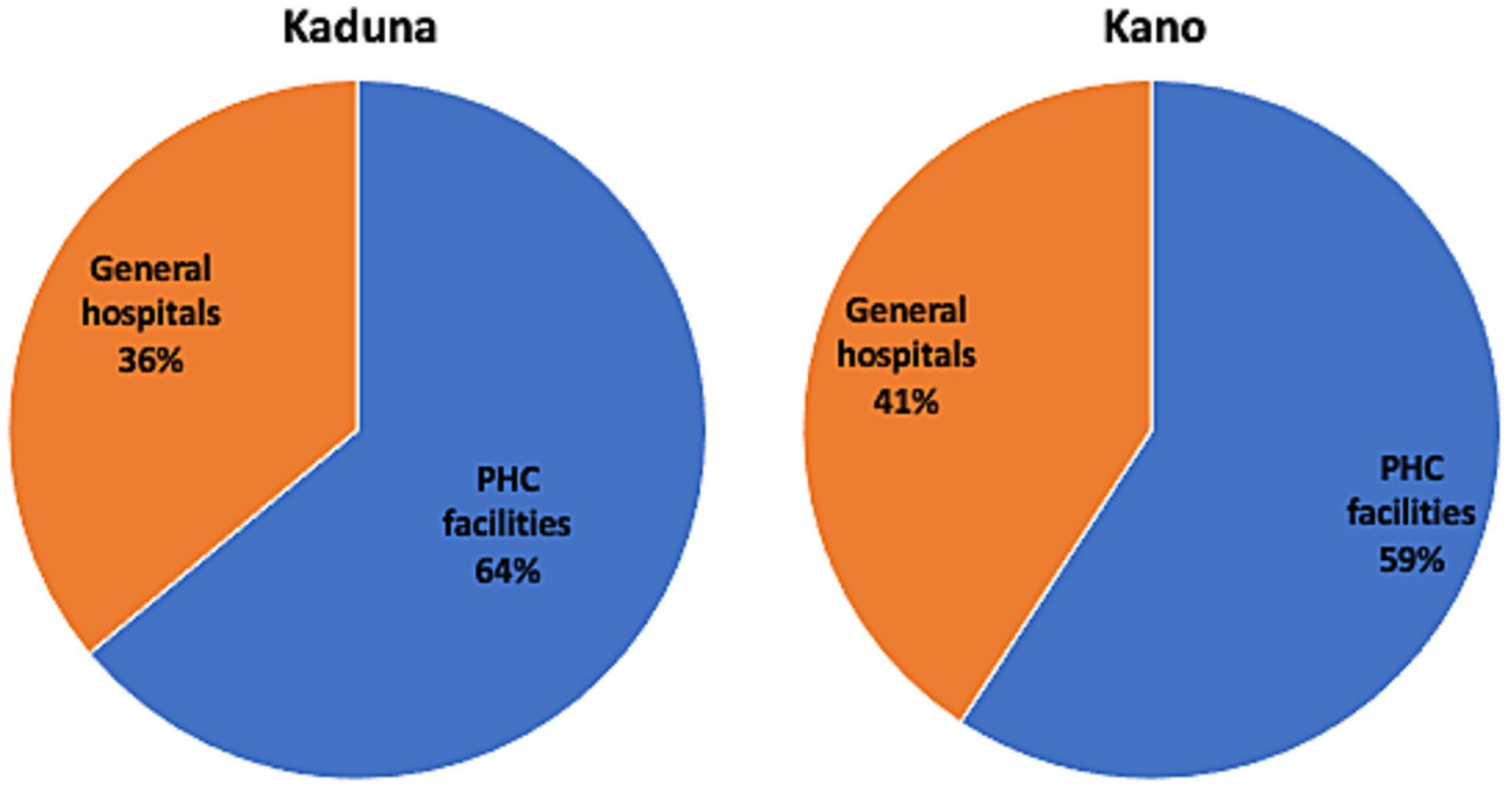
Distribution of PHC services at PHC facilities and general hospitals, Kaduna and Kano, DHIS2, 2019. PHC services are weighted service outputs. For PHC facilities, weighted service output is the sum of outpatient visits (OP) and 4.4 times inpatient (IP) days, where 4.4 is the ratio of the cost per IP day in primary hospitals (PPP I$ 21.69) over the cost per OP visit in bed-equipped health centers (PPP I$ 4.93), based on unit cost data from WHO-CHOICE (35). For general hospitals, it is the sum of OP visits and 3.9 times IP days, where 3.9 is the ratio of the cost per IP day in primary hospitals (PPP I$ 21.69) over the cost per OP visit in primary hospitals (PPP I$ 5.62).

In line with the national guidelines (4), which emphasize the pivotal role of PHC facilities in the delivery of PHC services, the results presented in Tables 3, 4 were derived considering only PHC facilities in Kaduna and Kano. However, data from the sampled general hospitals indicated that these hospitals provided large volumes of PHC services (Supplementary Table S3). Indeed, the DHIS2 data from 2019 showed that about a third of PHC services in both states were delivered at general hospitals (Figure 3 and Supplementary Table S4). Notably, partly owing to the higher volume of services provided, the average cost per patient at Kano’s general hospital PHC units was lower than that at PHC facilities (Supplementary Table S5).

To incorporate expenditures on PHC at general hospitals in the two states, we also calculated expanded total annual actual costs, extrapolating expenditures to both statewide PHC facilities and general hospitals. This adjustment slightly narrowed the gap between actual and normative costs from US$ 16.9–US$ 26 per capita to US$ 14.6–US$ 25.1 in Kaduna, and from US$ 29.1–US$ 33.6 to US$ 27.2–US$ 31.4 per capita in Kano (Table 5).

**Table 5:** Statewide actual and normative PHC costs per capita and corresponding gaps by input, at PHC facilities and general hospitals, Kaduna and Kano US$ 2019.

	Kaduna				Kano			
	Actual cost per capita		Normative cost per capita	Gap	Actual cost per capita		Normative cost per capita	Gap
	Estimate 1	Estimate 2			Estimate 1	Estimate 2		
Clinical labor	5.4	8.2	11.0	2.8–5.6	2.0	2.4	8.3	5.9–6.3
Drugs	9.4	14.4	19.4	5.0–10.0	10.3	12.8	20.3	7.5–10.0
Medical supplies	4.0	5.9	11.9	6.0–7.9	1.9	2.4	10.8	8.4–8.9
Indirect	1.3	2.0	2.9	0.9–1.6	3.5	4.4	9.8	5.4–6.3
Total	20.0	30.6	45.2	14.6–25.1	17.8	22.0	49.2	27.2–31.4
Number of PHC facilities and general hospitals in network				1,114				1,239
Population (million)				9.2				14.3

Actual cost per capita estimates calculated as follows: estimate 1, total actual cost in sample expanded to entire state based on service utilization, then divided by total state population; estimate 2, total actual costs in sample expanded to entire state based on numbers of facilities, then divided by total state population. Clinical labor in actual costs includes labor of doctor, nurse/midwife, community health officer, CHEW, JCHEW, CHIPS, health attendant, lab technician, pharmacy technician, environmental officer with clinical duties, pharmacist, lab scientist, nutritionist, health educator, CHIPS, other labor. Clinical labor in normative costs includes labor of doctor nurse/midwife, community health officer, CHEW, JCHEW, and CHIPS. Indirect costs in actual costs include operational expenses and labor of medical record officer, environmental officer and support volunteers. Indirect costs in normative costs are imputed based on the proportion observed in actual costs. General hospital costs in both states are based on Kano PHC units.

This analysis assumed that the whole population in each state relied only on public PHC services. However, more than half of the country’s health services are provided in the private sector (2). Modifying this assumption to 75% of the population in each state relying on public PHC services, would reduce the gaps between actual and normative costs at PHC facilities and general hospitals to US$ 3.3–US$ 13.8 per capita in Kaduna and US$ 14.9–US$ 19.1 per capita in Kano (Table 6). Further modification to 50% of the population relying on public PHC services yields mixed results for Kaduna: one estimate indicates a surplus in funding at PHC facilities and general hospitals, while the other indicates a gap of US$ 2.5 per capita. In contrast, the funding gap at public facilities in Kano would be reduced to between US$2.6 and US$6.8 per capita.

**Table 6:** Per capita gaps by 50 and 75% of population accessing PHC services at public PHC facilities and general hospitals, Kaduna and Kano US$ 2019.

	Kaduna		Kano	
	Per capita gaps by % of population accessing PHC services at public facilities		Per capita gaps by % of population accessing PHC services at public facilities	
	50%	75	50%	75%
Clinical labor	−2.7–0.1	0.0–2.8	1.8–2.2	3.8–4.2
Drugs	−4.7–0.3	0.2–5.2	−2.6–-0.2	2.5–4.9
Supplies	0.0–2.0	3.0–5.0	3.0–3.5	5.7–6.2
Indirect	−0.6–0.2	0.1–0.9	0.5–1.4	2.9–3.8
Total	−8.0–2.5	3.3–13.8	2.6–6.8	14.9–19.1

Clinical labor in actual costs includes labor of doctor, nurse/midwife, community health officer, CHEW, JCHEW, CHIPS, health attendant, lab technician, pharmacy technician, environmental officer with clinical duties, pharmacist, lab scientist, nutritionist, health educator, CHIPS, other labor. Clinical labor in normative costs includes labor of doctor nurse/midwife, community health officer, CHEW, JCHEW, and CHIPS. Indirect costs in actual costs include operational expenses and labor of medical record officer, environmental officer and support volunteers. Indirect costs in normative costs are imputed based on the proportion observed in actual costs. General hospital costs in both states are based on Kano PHC units.

## 4 Discussion

This study analyzed the actual and normative recurrent costs of PHC services at public health facilities in Kano and Kaduna, Nigeria. We found that average actual PHC costs per capita at PHC facilities—where most PHC services should be provided according to government guidelines (4)—ranged from US$ 18.9 to US$ 28 in Kaduna and US$ 15.9 to US$ 20.4 in Kano, contingent on the estimation methods used. When also considering the costs of PHC services provided at general hospitals—where approximately a third of PHC services are delivered in both states—the actual per capita costs of PHC services ranged from US$ 20 to US$ 30.6 in Kaduna and US$ 17.8 to US$ 22 in Kano. Notably, all estimates of actual PHC costs per capita were markedly lower than the normative per capita costs of delivering quality PHC services to all those who need them, projected at US$ 44.9 in Kaduna and US$ 49.5 in Kano.

Recent studies conducted in Afghanistan (38), Ethiopia (31), and Kenya (32) have similarly examined the actual and normative costs of PHC service packages. Consistent with our research, they revealed substantial resource gaps between existing PHC resources and the financial requirements to provide quality PHC services to all those who need them.

We were not able to identify any peer-reviewed studies examining the actual costs of PHC services in either Kaduna or Kano. Comparing state-level estimates with national figures for Nigeria is challenging since national data likely conceal substantial state-level variation. Per capita PHC cost estimates for Nigeria estimated by the Institute for Health Metrics and Evaluation (IHME) and the World Health Organization (WHO) ranged from US$ 31 in 2017 (43) to US$ 40 in 2020 (11). Although most of our estimates for Kaduna and Kano are lower, the IHME and WHO base their calculations on country-reported health expenditures that include components not considered in our study, including above service-level overheads and spending at private providers (43).

Comparisons of normative cost across different studies also pose challenges due to differences in methods, intervention packages, and target coverage levels. Nevertheless, our normative per capita costs align with the normative PHC cost estimates from the Disease Control Priorities 3^rd^ edition (DCP3) and the WHO, considering that both studies account for health systems costs and a wider intervention scope compared to the MSPs in Kaduna and Kano. The DCP3 reported a 2015 per capita cost of US$ 110 in lower middle-income countries for an Essential Universal Health Coverage (EUHC) package and a portion of the EUHC, the Highest-Priority Package (HPP), was costed at US$ 58 (44). The WHO’s 2030 projection sets the average per capita PHC cost at US$ 59 for lower middle-income countries (45).

In addition to the insights provided on the financial gap in Kaduna and Kano, data from this study also suggest that there are significant inefficiencies in the provision of PHC services at PHC facilities in both states. In Kaduna, the average costs per patient was US$ 82.2 ranging from US$ 9.4 to US$ 403.9. In Kano, it was US$ 133.6 ranging from US$ 28.2 to US$ 574.5. These average costs per patient are much higher than estimates of average cost per PHC service at PHC facilities for other countries in sub-Saharan Africa which typically range between US$ 5 and US$ 10 (46–48).

### 4.1 Policy implications

Our study results underscore the need to augment resources allocated to PHC services in Kaduna and Kano. Addressing the existing PHC financial resource gaps in the two states will require the concerted efforts of state governments as well as local governments and the federal government (2). The Lancet Global Health Commission on financing PHC stressed the need for pooling arrangements ensuring free PHC services at the point of care for all (34). In Nigeria, two important steps in this direction are (1) the establishment of the BHCPF (13) and (2) the recently enacted NHIA Act, which includes the establishment of a Vulnerable Group Fund—to be funded in part by the BHCPF—and mandates health insurance for all Nigerian citizens and legal residents, though the enforcement mechanisms for the mandate have yet to be delineated (49). Successful implementation of the NHIA Act is expected to significantly increase resources for PHC in all states (50).

Efforts to operationalize the NHIA Act are currently underway, which would directly or indirectly impact the design and operations of state health insurance schemes across Nigeria. There is therefore a unique opportunity to refine insurance processes and procedures to ensure that they foster the delivery of quality, efficient, and equitable primary health services in Nigeria. Understanding the costs associated with PHC services is vital to crafting provider payment strategies that assure fair compensation for providers, encouraging them to maintain high-quality service delivery (18–22). As per the recommendation of the Lancet Commission on financing PHC, under the NHIS—which was the precursor to the NHIA—accredited public and private healthcare providers received capitation payments for PHC (34). This provider payment mechanism for PHC services seems likely to persist across state health insurance schemes (51). Under the NHIS, these capitation payments amounted to N750/US$ 2.4 per member every quarter for a designated benefit package which closely aligns with the MSP and providers have criticized the payments as too low (18, 23, 24). Our study results on normative per capita costs could potentially inform the adjustment of capitation rates for PHC services across state health insurance schemes in Kaduna, Kano, and other states in Nigeria.

Beyond finances, addressing deep-seated causes of PHC inefficiencies is crucial (52). This study does not assess critical PHC service inputs like drug availability, system-level attributes like governance and leadership, and service delivery characteristics like quality of care and provider competence which are known to be significant issues in the Nigerian health system (2). The analysis does however shed light on some PHC inputs and processes and draws attention to significant inefficiencies in PHC service delivery in both states that should be considered in the context of the operationalization of the NHIA Act as well as the refinement of BHCPF operations.

The significant inefficiencies in the provision of PHC services at PHC facilities in both states appear to stem at least partly from an asymmetry between demand and supply at these facilities. On the supply side, we noted sizeable staffing discrepancies among PHC facilities in Kaduna and Kano, pointing to possible deviations from staffing guidelines and suggesting there is a need for better management of staff distribution across facilities. In addition, low levels of staff productivity also seem evident from our data on caseloads per clinical staff. We cannot rule out the possibility of errors in the service utilization and human resource data in our study. However, the low levels of productivity of clinical staff as reported by the DHIS2, correlates with other work conducted by the World Bank in Nigeria, which highlighted that factoring in a notably high staff absenteeism rate of 31.7% overall, the average caseload for clinical staff was 5.2 for all facilities—ranging from 2.3 at health posts to 5.6 at health centers (53).

On the demand side, the high average costs per patient we observed are partly due to low levels of utilization at PHC facilities. The 2019 DHIS2 data showed that about a third of PHC services in both states were delivered at general hospitals suggesting that many patients bypass PHC facilities in favor of higher-level facilities. Yet according to national guidelines, PHC services are meant to be provided mostly at PHC facilities (4). Bypassing can cause higher-level facilities to be overburdened, and PHC facilities to be underused (2). There is evidence that NHIS enrollees exhibited bypassing behavior due to quality concerns (54, 55). The extensive use of hospitals for PHC services underscores the need for a more effective referral system or gatekeeping measures, along with better patient education about the referral process. This bypassing of PHC facilities also underscores the need to strengthen accreditation processes of health facilities to deliver PHC services that ensure quality PHC services. The NHIA Act foresees that the NHIA will work with state health insurance schemes and Health Management Organizations (HMOs) to accredit public and private health facilities to deliver health services to enrollees (49). Research on the NHIS showed that more higher-level facilities tended to be accredited than PHC facilities.

### 4.2 Limitations

These study results provide valuable insights into the actual costs of PHC services in Kaduna and Kano at the facilities delivering these services, as well as the costs of providing the services delineated in the MSPs to all those who need them. However, several limitations should be kept in mind when considering our findings. First, we acknowledge that there are limitations associated with our actual costs. Facility data on drugs and medical supplies were supplemented with costing based on estimated consumption patterns, introducing potential biases. Our assessment of the PHC costs at general hospitals focused on the costs incurred by PHC units and this focus likely resulted in an underestimation of the costs of delivering PHC services at these hospitals. The use of data from Kano as a proxy for Kaduna may also have resulted in an underestimation of costs, given the variations in the cost structures of PHC facilities between the two states. Additionally, in extrapolating from a non-random sample of public PHC facilities and general hospitals to all public PHC facilities and general hospitals in Kaduna and Kano, we assumed that the average PHC service costs in the sampled facilities were representative of costs for all facilities. Because of some of the sample selection criteria including health data reporting, facility accessibility, and security, it is probable that better quality health facilities dominated the sample. This likely skewed the actual costs upward, leading to an underestimation of the gap between actual and normative costs. The merging of PHC facilities into a single category due to classification discrepancies between the Master Facility List and on-ground observations may also have influenced the results. Second, the study relied on DHIS2 service utilization data which have known issues with data quality and completeness (2). Third, we used STPs developed by a team of clinicians to estimate normative costs which may have introduced certain biases. Considerable effort was invested in creating population in need estimates, but we encountered data constraints (2). When incidence or prevalence data for only one state was available, it was used for both states. In cases where state-specific data were not available, national, or international incidence or prevalence estimates were used instead. Of note, normative costs included only point of care diagnostics and excluded laboratory costs, indirect costs were based on data collected in the actual costing, and the normative costs did not consider potential efficiency gains through economies of scale and scope. It is important to note that from the outset, the study was designed to analyze recurrent costs at public PHC facilities, intentionally omitting capital and above-facility from both the actual and normative cost estimates. Although such exclusions are not uncommon in many costing studies (56), it implies that both actual and normative costs associated with public facilities are likely underestimated. Specifically, while the study’s short-term framing makes the exclusion of capital reasonable, the need for capital investments in PHC in Nigeria is well documented (2). In addition, the OOP expenditures captured focused on patient expenses on drugs and medical supplies at facilities and do not capture the full range of OOP expenses incurred. Finally, although the quality of PHC services is vital to an effective PHC system (52) and may correlate with costs (57), our study did not evaluate the quality of actual PHC service provision, or the resources required to improve quality of care.

### 4.3 Future research

Even if the study limitations make the study results more indicative than definitive (29), they serve as an important baseline for understanding current investment in PHC in Nigeria. For more comprehensive insight into the costs and service provision of PHC in Nigeria, additional data collection would be beneficial. This should include detailed actual cost data by service category, comprehensive data on OOP PHC expenses, service coverage and cost data from private providers, faith-based organizations, and NGOs, service quality data, and human resource data that provides insights on absenteeism and idle staff time.

Reliable electronic data sources would make costing studies easier to implement, reducing the time necessary for data collection (i.e., surveys) and allowing for more real-time cost analyses. Updated facility lists and corresponding catchment population data are needed at both state and national levels. There is a pressing need for more transparency on cost and expenditure data necessary for improved decision-making. The implementation of logistics management information systems, drug expenditure tracking, electronic point-of-sale systems, electronic medical records, and human resource information systems would facilitate accurate tracking and help address issues related to health worker distribution, shortages, excesses, and skill mix imbalances.

### 4.4 Conclusion

Despite efforts to improve public PHC services in Kaduna and Kano, a discernable financial gap exists between current resources available for PHC services and estimated normative costs. Our data suggests that while increased resources are pivotal to bridging this gap, improving the efficiency of current PHC expenditures in both states is also critical. The study’s insights on normative per capita costs could serve as valuable inputs for modifying capitation rates for PHC services not only in Kano and Kaduna but also in other states in Nigeria.

## Data availability statement

The raw data supporting the conclusions of this article will be made available by the authors, without undue reservation.

## Author contributions

CG and YO designed the study. YO, UE, NK, and PO led data collection, validation, and interpretation. RM, NK, and MO conducted data analysis on costs and service utilization. HA, TH, and DW provided critical revisions and inputs on the relevance of the findings to policy reforms. MO, CG, RM, and EL wrote the first draft of the manuscript. All authors interpreted the data and contributed to the manuscript. Aside from CG and YO, authors are listed in alphabetical order.

## Footnotes

^1^4.4 represents the ratio of the cost per inpatient day at primary hospitals international dollar (PPP I$) 21.69 to the cost per outpatient visit at health centers with beds (PPP I$ 4.93) and 3.9 represents the ratio of the cost per inpatient day at primary hospitals (PPP I$ 21.69) to the cost per outpatient visit at primary hospitals (PPP I$ 5.62) based on unit cost data from WHO-CHOICE.
