# Supplementary material for: An assessment of primary health care costs and resource requirements in Kaduna and Kano, Nigeria
Source: Front Public Health. 2023 Dec 19;11:1226145. doi: 10.3389/fpubh.2023.1226145 (PMC10794985; doi:10.3389/fpubh.2023.1226145)
Supplement: Supplementary file 1 [file Table_1.docx]

Supplementary Material

An assessment of primary health care costs and resource requirements in Kaduna and Kano, Nigeria

Yewande Ogundeji, Hamza Abubakar, Uche Ezeh, Tijjani Hussaini, Nelson Kamau, Eliza Love, Rodrigo Muñoz, Paul Ongboche, Marjorie Opuni, Damian G. Walker, Colin Gilmartin^*^

*** Correspondence:** Colin Gilmartin cgilmartin@msh.org

## Supplementary Figures

**Supplementary Figure S1. Overview of Minimum Standards for PHC in Nigeria**

| **Communicable diseases**   - HIV prevention and treatment - Malaria prevention and treatment - TB screening and treatment - STI screening and treatment - Measles treatment - Whooping cough treatment - Treatment of respiratory infections - Leprosy treatment and support - NTD screening, diagnosis, and treatment - Other communicable diseases | **Maternal and newborn care**   - Antenatal care - Skilled delivery care - Postnatal care - Neonatal care | **Non communicable diseases**   - Anemia diagnosis and treatment - Asthma management - Care for minor accidents - Cardiovascular screening - Diabetes screening - Hypertension screening - Arthritis screening - Treatment of eye conditions - Ear, nose, throat care - Oral health - Mental health screening/counseling |
| --- | --- | --- |
| **Family planning**   - Counselling - Dispensing of contraceptives | **Child survival**   - Immunization - Integrated management of childhood illness | **Nutrition**   - Nutrition screening - Management of malnutrition - Promotion of proper nutrition and food education |
| **Health education and community mobilization**   - IEC and BCC - Community mobilization - Home visits and community outreach |  |  |

**Supplementary Figure S1.** Source: National Primary Health Care Development Agency (2016) Minimum Standards for PHC in Nigeria. Available at http://www.nphcda.gov.ng/

**Supplementary Figure S2. Overview of benefit package, National Health Insurance Scheme**

| **Out-patient care** (including consumables)   - Proper history taking, examination, routine laboratory investigations | **Routine immunization**   - Immunization against childhood killer diseases | **Surgical procedures**   - Drainage of simple abscess - Minor wound debridement - Surgical repairs of simple lacerations - Drainage of paronychia - Circumcision of male infants - Passage of urethral catheter |
| --- | --- | --- |
| **Internal medicine**   - Malaria and other acute uncomplicated febrile illnesses - Uncomplicated Diarrheal diseases - Acute upper respiratory tract infections - Uncomplicated pneumonia - Simple anemia (not requiring blood transfusion) - Simple skin diseases - Worm infestation - Other uncomplicated bacteria, fungal, parasitic and - viral infections and illnesses - Dog bites, snakebites, scorpion stings - Arthritis | **HIV/AIDS**   - Counseling and testing - Health education - Treatment of simple opportunistic infections | **STIs**   - Counseling - Health Education - Management of uncomplicated STIs |
| **Mental Health**   - Psychosomatic illnesses - Insomnia - Other | **Pediatrics**   - Feeding problems and nutritional services - Treatment of common childhood illnesses | **Obstetrics and Gynecology**   - Acute pelvic inflammatory diseases - Vaginal discharges - Routine maternity care for all pregnancies - Post natal care cover the neonate and preterm/ premature babies |
| **Ophthalmology**   - Conjunctivitis - Simple contusion, abrasions, foreign bodies etc. | **Emergency care**   - Establishing an intravenous line - Establishing patent airway - Management of convulsion - Control of bleeding - Cardio-pulmonary resuscitation - Immobilization of fractures using splints, neck collars, to ease transportation of patients - Aspiration of mucus plug to clear airways - Asthmatic Attacks - Any other procedure that may be life saving | **Family planning**   - Family planning education only |
| **Child welfare services**   - Growth monitoring - Routine immunization - Nutritional advice and health education | **Dental Care**   - Dental care education only |  |

**Supplementary Figure S2.** Source: National Health Insurance Scheme. National health insurance scheme 2012 operational guidelines, 2012. Available at https://www.dhmlnigeria.com/upload/NHIS_OPERATIONAL_GUIDELINES(Revised)%20(1).pdf

**Supplementary Figure S3. Map of sampled local government areas in Kaduna and Kano**


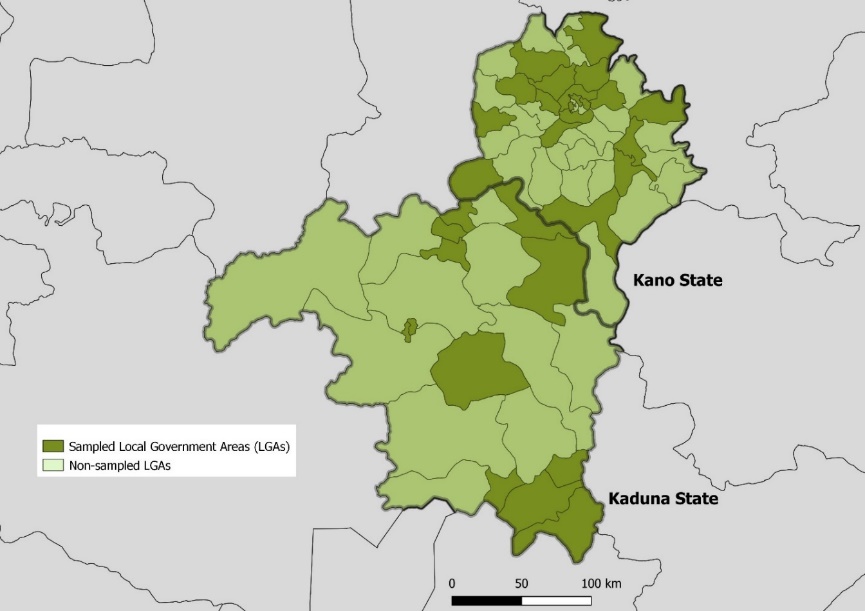


## Supplementary Tables

**Supplementary Table S1. Sample of health facilities by selected local government areas in Kaduna and Kano**

| **State** | **Local government area** | **Health post** | **Health clinic** | **Health center** | **General hospital** |
| --- | --- | --- | --- | --- | --- |
| **Kaduna** | Zaria |  |  | 1 | 1 |
|  | Sabon Gari |  | 2 | 1 | 1 |
|  | Kubau |  | 1 | 1 |  |
|  | Ikara |  | 1 |  |  |
|  | Kudan |  | 1 |  |  |
|  | Jaba |  | 1 |  | 1 |
|  | Jema'a |  | 1 |  |  |
|  | Kaura |  |  | 1 |  |
|  | Sanga | 1 |  |  |  |
|  | Kauru |  | 2 | 2 | 1 |
|  | Kaduna North |  |  | 2 | 1 |
|  | Kaduna South |  |  | 2 |  |
|  | Total | 1 | 9 | 10 | 5 |
| **Kano** | Ajingi |  | 1 | 1 |  |
|  | Bagwai |  | 1 |  |  |
|  | Bichi | 1 |  |  | 1 |
|  | Danbata |  |  | 1 |  |
|  | Dawaki Tofa |  | 1 |  |  |
|  | Fagge |  |  |  | 1 |
|  | Garko | 1 |  |  |  |
|  | Gezawa |  |  | 1 |  |
|  | Gwale |  | 1 |  |  |
|  | Gwarzo |  |  | 1 |  |
|  | Kano | 1 |  | 1 |  |
|  | Kumbotso | 1 |  | 1 |  |
|  | Madobi |  | 1 |  |  |
|  | Minjibir |  | 1 |  |  |
|  | Nasarawa |  |  |  | 1 |
|  | Rogo | 1 |  |  |  |
|  | Tofa | 1 |  |  |  |
|  | Tudun Wada |  | 2 |  |  |
|  | Ungogo | 1 |  |  |  |
|  | Wudil |  |  |  | 1 |
|  | Total | 7 | 8 | 6 | 4 |

**Supplementary Table S2. Normative costs by PHC service, Kaduna and Kano**

| **Normative PHC service** | **Kaduna** | | **Kano** | |
| --- | --- | --- | --- | --- |
|  | **Total cost (US$)** | **Cost per case (US$)** | **Total cost (US$)** | **Cost per case (US$)** |
| 101. Antenatal | 8,529,378 | 18.00 | 11,678,068 | 15.85 |
| 102. Diagnose/treat malaria | 549,927 | 7.40 | 979,382 | 8.50 |
| 103. Diagnose/Treat severe malaria | 310,030 | 21.87 | 466,299 | 21.21 |
| 104. Diagnose/treat anemia | 1,079,340 | 3.73 | 1,052,730 | 2.34 |
| 105. PMTCT/HIV-no cost ARV | 2,175,641 | 270.60 | 3,369,133 | 270.20 |
| 106. Treatment of Syphilis | 128,939 | 16.93 | 196,532 | 16.64 |
| 107. Treat other STDs apart from syphilis | 164,252 | 6.47 | 270,217 | 6.86 |
| 108. Basic obstetric care (normal delivery) | 4,379,600 | 12.49 | 6,788,016 | 12.45 |
| 109. Provide Vit A to post-partum woman | 95,856 | 3.31 | 140,846 | 3.13 |
| 110. Emergency obstetric care: post-abortion care | 638,699 | 12.22 | 930,207 | 11.48 |
| 111. Labor complications (Severe pre-eclampsia/Eclampsia) - Pre-referral treatment | 87,225 | 15.27 | 121,388 | 13.70 |
| 112. Labor complications (Post-partum Hemorrhage - heavy bleeding): Pre-referral treatment | 269,922 | 22.78 | 338,604 | 18.43 |
| 113. Labor complications (obstructed labor) - Pre-referral treatment | 170,042 | 3.23 | 227,822 | 2.79 |
| 114. Labor complications (Sepsis) - Pre-referral treatment | 327,576 | 49.94 | 405,054 | 39.82 |
| 115. Postnatal care | 3,455,980 | 9.86 | 4,373,406 | 8.02 |
| 201. Manage neonatal tetanus (refer to next level SCBU) | 498,439 | 1.42 | 7,567 | 1.16 |
| 202. Initiation of early breastfeeding (within 30mins after birth) | 124,610 | 0.36 | 157,656 | 0.29 |
| 203. Prevent new-born infection (e.g., using chlorhexidine gel) | 508,962 | 1.45 | 683,175 | 1.25 |
| 204. Prevent and manage new-born hypo/hyperthermia | 6,026 | 0.07 | 7,624 | 0.06 |
| 205. Early asphyxia identification and management | 5,240 | 0.45 | 7,911 | 0.44 |
| 206. Prevent and manage ophthalmic neonatorum | 3,240 | 0.84 | 4,243 | 0.71 |
| 207. Identification and management of sick new-born (sepsis) | 116,217 | 7.76 | 121,733 | 5.23 |
| 208. Care of preterm and/or low birth weight new-born | 62,666 | 0.66 | 75,829 | 0.51 |
| 301. Identification of eligible pregnant women and children | 345,031 | 0.98 | 312,383 | 0.57 |
| 302. Immunization services TD, BCG, OPV, DPT, YF, MMR etc. | 17,250,915 | 49.22 | 26,372,040 | 48.37 |
| 303. Immunization trend follow up | 206,575 | 0.59 | 216,755 | 0.40 |
| 304. Adverse effect following immunization (AEFI) | 92,951 | 5.30 | 119,438 | 4.38 |
| 305. Assist in the identification of acute flaccid paralysis (AFP) | 3,831 | 0.16 | 4,676 | 0.13 |
| 306. Monitor ORT/ feeding for diarrhea; | 677,364 | 3.60 | 825,390 | 2.80 |
| 307. Pneumonia treatment | 261,096 | 1.39 | 255,910 | 0.87 |
| 308. Treat ARI with antibiotics; | 188,916 | 1.00 | 193,913 | 0.66 |
| 309. Treat measles | 25,162 | 0.68 | 36,548 | 0.63 |
| 310. Treatment of Malaria | 6,240,154 | 4.76 | 7,214,412 | 3.51 |
| 401. Counselling and motivation for FP | 1,519,334 | 1.03 | 1,918,208 | 0.84 |
| 402. Dispensing of male and female condoms | 8,191,747 | 3.82 | 12,216,342 | 3.67 |
| 403. Dispensing of Oral contraceptives | 400,812 | 5.37 | 587,780 | 5.07 |
| 404. Administering injectables | 5,005,216 | 33.32 | 7,556,195 | 32.43 |
| 405. HCT and lab tests | 466,343 | 0.71 | 588,226 | 0.58 |
| 406. Insert implants | 18,516,803 | 118.59 | 28,478,192 | 117.60 |
| 407. Insert IUD | 435,176 | 11.62 | 583,341 | 10.04 |
| 501. HIV testing Services (HTS) | 19,094,016 | 5.00 | 5,849,328 | 3.95 |
| 502. Initiate anti-retroviral therapy (ART >10 years) | 29,205,393 | 279.38 | 45,189,058 | 278.78 |
| 503. Initiate anti-retroviral therapy (ART<10 years) | 404,526 | 117.46 | 630,121 | 116.85 |
| 504. Initiate anti-retroviral (ARV) for pregnant women | 40,447 | 5.03 | 57,684 | 4.63 |
| 505. Syndromic management of STIs | 9,485,273 | 11.01 | 15,211,919 | 11.39 |
| 601. TB testing | 5,535,912 | 1.93 | 6,668,054 | 1.50 |
| 602. Initiate TB treatment in adults (>25kg) | 331,626 | 30.59 | 492,381 | 29.29 |
| 603. Initiate TB treatment in children (<25kg) | 62,284 | 36.17 | 122,679 | 45.50 |
| 701. Malaria Prevention- Provide LLINs | 1,279,340 | 0.68 | 1,724,710 | 0.58 |
| 702. Malaria diagnosis | 4,349,331 | 2.23 | 6,404,114 | 2.12 |
| 703. Treatment of uncomplicated malaria | 6,675,366 | 3.85 | 9,732,810 | 3.62 |
| 704. Treatment of severe malaria (Pre-referral management) | 17,431,800 | 80.87 | 27,367,361 | 81.80 |
| 801. Vaccinate Hepatitis B negative individuals | 11,096,310 | 17.23 | 16,079,804 | 16.05 |
| 802. Screening and diagnosis of hepatitis infection | 5,311,813 | 2.31 | 3,894,536 | 1.09 |
| 803. Life course vaccine for adolescents and adult (HBV/HPV) | 314,584 | 0.56 | 379,981 | 0.43 |
| 901. Preventive chemotherapy | 6,953,532 | 1.07 | 8,778,571 | 0.87 |
| 902. Screening and diagnosis of NTDs | 4,100,005 | 0.63 | 5,852,378 | 0.58 |
| 903. Provide treatments for cases of NTDs | 4,635,684 | 0.71 | 5,852,378 | 0.58 |
| 1101. Screening and diagnosis of EPDs | 13,580,827 | 1.42 | 17,182,429 | 1.16 |
| 1102. Provide treatments for cases of EPDs | 21,729,324 | 2.28 | 27,491,887 | 1.85 |
| 1201. Identification of cases of minor ailments | 470,802 | 0.49 | 569,696 | 0.38 |
| 1202. Provide treatments of minor ailments | 1,945,378 | 2.04 | 1,890,067 | 1.27 |
| 1301. Provision of micronutrients, vitamin A supplementation for children | 365,356 | 0.19 | 442,365 | 0.15 |
| 1302. Management of moderate malnutrition | 3,841,165 | 4.25 | 6,320,969 | 3.76 |
| 1303. Management of severe malnutrition | 36,000,193 | 86.65 | 75,975,818 | 85.84 |
| 1304. Food demonstration | 445,770 | 0.49 | 644,850 | 0.38 |
| 1305. Deworming for under-5s | 1,070,998 | 0.57 | 1,589,064 | 0.54 |
| 1306. Nutrition screening | 1,510,435 | 0.80 | 1,979,687 | 0.67 |
| 1307. Promotion of exclusive breastfeeding | 214,883 | 0.61 | 179,762 | 0.33 |
| 1308. promotion of use of iodized salt | 776,903 | 0.37 | 1,133,006 | 0.35 |
| 1309. promotion of dietary diversification | 208,601 | 0.10 | 251,652 | 0.08 |
| 1310. Complications of worm infestation | 14,019,844 | 6.63 | 14,830,082 | 4.52 |
| 1401. IEC necessary behavior change messages on prevailing health issues, problems, and prevention | 746,946 | 0.39 | 632,370 | 0.21 |
| 1403. Community mobilization for health | 746,946 | 0.39 | 632,370 | 0.21 |
| 1404. Home visits and community outreach | 497,964 | 0.26 | 421,580 | 0.14 |
| 1502. Screening for sore throat, fever, and joint pains to rule out acute rheumatic fever | 282,317 | 0.82 | 339,463 | 0.64 |
| 1503. Counselling on lifestyle management based on findings from the risk assessment | 225,905 | 0.66 | 271,632 | 0.51 |
| 1504. Commence aspirin in individuals with high risk of having an adverse cardiovascular event | 2,280,772 | 6.64 | 2,961,429 | 5.57 |
| 1506. Referral to secondary/tertiary health facilities for further management | 282,381 | 0.82 | 339,540 | 0.64 |
| 1507. Support for self-management | 112,952 | 0.33 | 135,816 | 0.26 |
| 1601. Urgent and facilitated referral through an escort if BP >180/>110 mm Hg | 990,948 | 4.77 | 3,664,133 | 11.35 |
| 1602. Refer if SBP ≥140 or DBP ≥ 90 mmHg in people < 40 yrs. (to exclude secondary hypertension) | 370,521 | 0.58 | 1,256,679 | 1.27 |
| 1603. Initiate drug treatment if SBP ≥130 or ≥ DBP 90 mmHg with diabetes | 144,981 | 4.17 | 174,476 | 3.23 |
| 1604. Initiate lifestyle management if SBP ≥120 or DBP ≥ 80 mmHg | 1,641,566 | 1.67 | 1,592,394 | 1.04 |
| 1605. Commence drug treatment if persistent BP ≥140/90 mm Hg | 1,619,157 | 1.64 | 2,886,034 | 1.89 |
| 1606. Support for self-management and care (e.g., regular blood pressure monitoring) | 47,078,138 | 47.80 | 72,973,449 | 47.70 |
| 1701. Screening for various types of diabetes | 405,719 | 0.39 | 570,148 | 0.35 |
| 1702. Pre-referral treatment for Hyperglycemic Hyperosmolar sickness (Diabetic complications) | 3,029,235 | 24.72 | 4,441,499 | 23.35 |
| 1801. Screening and examination | 8,502,582 | 16.43 | 13,267,490 | 16.34 |
| 1802. Promotion of self-Breast Examination | 85,054 | 0.16 | 103,783 | 0.13 |
| 2001. Treatment of minor eye infections with topical eye drugs | 313,868 | 0.33 | 379,797 | 0.26 |
| 2002. Allergic Conjunctivitis/Foreign body in eyes | 2,934,092 | 3.07 | 7,417,645 | 4.99 |
| 2003. Infective conjunctivitis/ Pustule in the eyelid | 4,692,327 | 4.91 | 11,025,392 | 7.42 |
| 2101. Screening and examination of COPD | 8,471 | 0.33 | 10,225 | 0.26 |
| 2102. Management of COPD | 167,674 | 6.50 | 289,797 | 7.24 |
| 2103. Management of asthma | 1,830,758 | 5.34 | 3,707,839 | 6.95 |
| 2104. Support for self-management and care of asthma | 571,411 | 1.67 | 555,164 | 1.04 |
| 2201. Identification and screening of mental disorders | 2,680,726 | 3.06 | 2,604,506 | 1.91 |
| 2202. Management of depression | 358,126 | 1.39 | 347,944 | 0.87 |
| 2203. Management of epilepsy | 69,627 | 1.58 | 59,938 | 0.88 |
| 2205. Management and treatment of Dementia cases | 22,812 | 2.04 | 22,107 | 1.27 |

**Supplementary Table S3. PHC services at sampled PHC facilities and general hospitals in Kaduna and Kano, 2019**

|  |  | **Kaduna** |  |  | **Kano** |
| --- | --- | --- | --- | --- | --- |
|  | **PHC facility** | **General hospital** |  | **PHC facility** | **General hospital** |
| **Number of sampled facilities** | 21 | 4 |  | 21 | 3 |
| **Outpatient visits per year** |  |  |  |  |  |
| Mean | 2,483 | 13,807 |  | 3,006 | 42,768 |
| Median | 2,086 | 11,136 |  | 1,924 | 20,400 |
| Range | 608–7,553 | 3,131–29,824 |  | 425–13,034 | 3,469–104,436 |
| **Inpatient days per year** |  |  |  |  |  |
| Mean | 114 | 8,540 |  | 0 | 9,690 |
| Median | 5 | 4,877 |  | 0 | 5,239 |
| Range | 0–576 | 917–23,488 |  | 0 | 596–23,234 |

**Supplementary Table S4. PHC services delivered at PHC facilities and general hospitals in Kaduna and Kano, 2019**

|  | **Kaduna** | | |  | **Kano** | | |
| --- | --- | --- | --- | --- | --- | --- | --- |
|  | **PHC facility** | **General hospital** | **Total** |  | **PHC facility** | **General hospital** | **Total** |
| **Actual number of PHC services** |  |  |  |  |  |  |  |
| Outpatient visits | 1,907,965 | 421,013 | 2,328,978 |  | 2,830,690 | 1,310,591 | 4,141,281 |
| Inpatient days | 61,085 | 206,680 | 267,765 |  | 55,462 | 206,680 | 500,030 |
|  |  |  |  |  |  |  |  |
| **Number of facilities in network** | 1,080 | 34 | 1,114 |  | 1,207 | 32 | 1,239 |
| **Population (million)** |  |  | 9.2 |  |  |  | 14.3 |

**Supplementary Table S5. Total PHC costs and costs per patient at sampled general hospitals in Kano, US$ 2019**

| **Number of sampled facilities** | 3 |
| --- | --- |
|  |  |
| **Total costs** |  |
| Mean | 787,500 |
| Range | 661,811–907,142 |
|  |  |
| **Cost per patient** |  |
| Mean | 50 |
| Range | 5–138 |
